# A Lightweight and Privacy-Friendly Data Aggregation Scheme against Abnormal Data

**DOI:** 10.3390/s22041452

**Published:** 2022-02-14

**Authors:** Jianhong Zhang, Haoting Han

**Affiliations:** 1 School of Information Sciences and Technology, North China University of Technology, Beijing 100043, China; ncuthht105@mail.ncut.edu.cn; 2 Guizhou Provincial Key Laboratory of Public Big Data, Guizhou University, Guiyang 550025, China

**Keywords:** data aggregation, abnormal data, source, matrix encryption, lightweight

## Abstract

Abnormal electricity data, caused by electricity theft or meter failure, leads to the inaccuracy of aggregation results. These inaccurate results not only harm the interests of users but also affect the decision-making of the power system. However, the existing data aggregation schemes do not consider the impact of abnormal data. How to filter out abnormal data is a challenge. To solve this problem, in this study, we propose a lightweight and privacy-friendly data aggregation scheme against abnormal data, in which the valid data can correctly be aggregated but abnormal data will be filtered out during the aggregation process. This is more suitable for resource-limited smart meters, due to the adoption of lightweight matrix encryption. The automatic filtering of abnormal data without additional processes and the detection of abnormal data sources are where our protocol outperforms other schemes. Finally, a detailed security analysis shows that the proposed scheme can protect the privacy of users’ data. In addition, the results of extensive simulations demonstrate that the additional computation cost to filter the abnormal data is within the acceptable range, which shows that our proposed scheme is still very effective.

## 1. Introduction

With the application of electricity in our daily life becoming increasingly extensive, more factors need to be considered in the production decisions of the cloud server [1,2], such as how to maintain a balance between supply and demand when electricity usage changes dramatically [3]. Thus, it is critical to obtain the electricity usage data of all users. In addition the smart grid, as a key infrastructure, adds upstream information feedback based on the traditional grid, which can help us collect the electricity usage data of users in various regions [4,5]. The prominent advantage of smart meters is to make sure that electricity supply matches the demand of users within a short period, which is of great significance for the rational distribution of power resources and the reduction of economic losses [6,7]. To obtain the real-time electricity demand of users, their electricity usage data should be measured, aggregated, and analyzed through advanced metering infrastructure [8,9].

However, it is a noteworthy problem of the smart grid that the abnormal electricity data, caused by electricity theft or meter failure, can lead to inaccurate aggregation results. This not only harms the personal interests of users, but also interferes with the production decisions of the cloud center. To the best of our knowledge, none of the existing schemes consider the impact of abnormal data. In the extant schemes, the aggregation center is responsible for aggregating all the reported electricity usage data of smart meters but cannot detect whether the reported data is abnormal, let alone find the source of the abnormal data.

Therefore, it is an important challenge to filter out the abnormal data and find the source of the abnormal data when the data is encrypted. To address this issue, we propose a lightweight and privacy-friendly data aggregation scheme against abnormal data, in which the valid data is correctly aggregated, but the abnormal data is automatically filtered out during the aggregation process. Notably, the filtration of the abnormal data does not need additional procedures, which is the highlight of this work. Besides, compared with other methods in other schemes, the encryption method used in our scheme is more suitable for smart meters with limited computing capacity. Specifically, the main contributions of this paper are summarized as follows:

- We propose a lightweight and privacy-friendly data aggregation scheme against abnormal data by using lightweight matrix encryption. It is suitable for smart meters with limited computing power, since no time-consuming computation operators are involved.
- Abnormal data can automatically be filtered out without additional procedures. In addition, the source of the abnormal data can also be found out in this process. Thereby, accurate aggregation results can be obtained through the proposed scheme, and abnormal meters can also be identified for maintenance, even if the data is encrypted.
- Finally, a detailed security analysis is provided to prove that our scheme can fully ensure the privacy and security of users’ data. Experiments and performance evaluations demonstrate that our scheme has a low computation cost and high practicality.

The rest of the paper is outlined as follows. In Section 2, some related works are provided. The preliminary is provided in Section 3. Section 4 illustrates the system model and adversary model. We propose the details of our scheme in Section 5, followed by the security analysis of our scheme in Section 6. A performance analysis is conducted in Section 7. Finally, the conclusion of our scheme is summarized in Section 8.

## 2. Related Work

There exist extensive data aggregation schemes on the topic of protecting users’ privacy in smart grids [10,11,12,13,14,15,16,17,18,19,20,21,22,23]. Homomorphic encryption has been applied in several works to achieve privacy-preserving data aggregation [10,11,12,13,14,15,16,17,18,19]. Shen et al. [10] proposed a Paillier-based data aggregation scheme against malicious data mining attacks, which can prevent the adversary from inferring a target user’s electricity usage data and obtain accurate aggregated results of electricity usage data. Xue et al. [11] proposed a privacy-preserving service-outsourcing scheme for a real-time pricing demand response in a smart grid, which solves the privacy issues by modifying the Paillier cryptosystem to hold two different decryption keys and achieves the flexible enrollment and revocation of smart meters. In addition, Saleem et al. [12] proposed a scheme to resist the malfunctioning of smart meters for data aggregation based on a modified Paillier cryptosystem. Their system can resist false data injection attacks by filtering out the inserted values from external attackers. For achieving secure data aggregation, the ElGamal-based algorithm has been taken into account [13,14]. Liu et al. [14] proposed a lifted elliptic ElGamal-based privacy-preserving data aggregation scheme, in which the trusted third party is removed and the users, with some measure of trust, construct a virtual aggregation area to mask the single user’s data against the denial of service attack. In order to resist quantum attacks and improve the efficiency of the algorithm, the lattice-based homomorphic approach has been applied to achieve secure data aggregation for smart grids [15,16]. Abdallah et al. [16] proposed a lattice-based privacy-preserving data aggregation scheme for a smart grid, which can further reduce the computation burden for smart appliances, because it depends on simple arithmetic operations. In [17], a privacy-friendly data aggregation scheme is proposed by Vahedi et al. They use elliptic curve digital signature algorithms (ECDSA) in smart grids to protect users’ privacy from the grid operators. Besides, to meet the higher data analysis requirements of the cloud server, multidimensional data is aggregated in some schemes [18,19,20]. Although the schemes based on homomorphic encryption can obtain accurate aggregation results, a heavy computational and communication burden will also be imposed on smart meters with limited computing power.

As another major encryption technology, masking-value-based schemes also have been proposed to achieve secure and efficient data aggregation in smart grids. As for masking-based data aggregation schemes [21,22,23,24], Gope et al. [21] first proposed a lightweight and privacy-friendly masking-based spatial data aggregation scheme for secure forecasting of power demands in smart grids. Their scheme only uses lightweight cryptographic primitives, such as exclusive OR operations and hash functions, thus it has a significantly lower computational cost as compared with other approaches. The LCEDA scheme proposed by Su et al. [22] achieves an efficient update of masking the value share to ensure forward security of individual data, dynamic enrollment, and revocation of smart meters. Moreover, Huang et al. [23] propose a lightweight and fault-tolerable data aggregation scheme that can determine the smart meters which fail to upload data on time with the idea of flag bit, and correct aggregation results can be obtained even if the data is not reported by the smart meters. However, the existing masking-based aggregation schemes cannot screen abnormal electricity consumption data either.

In addition, accurate aggregation results can be obtained by utilizing zero-knowledge proof [24], but heavy communication and the computational burden will also be imposed on the smart meters with limited computing power. Thus, the solution using zero-knowledge proof is not practical.

Therefore, we propose a lightweight and privacy-friendly data aggregation scheme against abnormal data by using matrix encryption, which can effectively filter abnormal data and find out the source of abnormal data. To more intuitively show the advantages of the proposed scheme compared with other schemes, the security feature comparisons are shown in Table 1.

## 3. Preliminaries

In this section, the preliminaries of the proposed scheme are presented, in which we describe the basic idea of filtering abnormal data.

**Figure 1:**
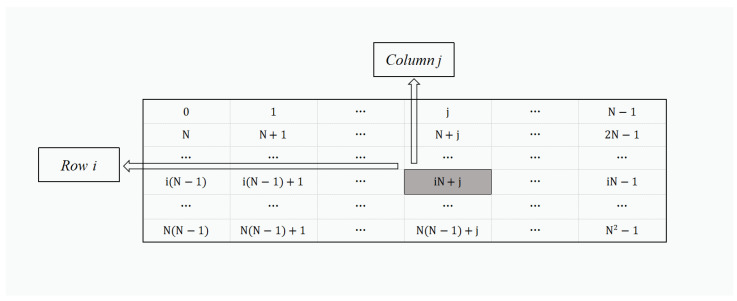
Representation of the constructed matrix.

***Filtering abnormal data***: Suppose that ***a*** is the data to be determined and ***b*** is the upper limit of the normal value, and they are in the range of $\left[0,N^{2}-1\right]$. Then, whether the data ***a*** is abnormal can be determined as follows [25]:

1. Construct an N×N matrix containing all possible values in $\left[0,N^{2}-1\right]$, as shown in Figure 1. Each value has a row coordinate and a column coordinate in this matrix.
  The value in the matrix can be represented by *iN + j*, the corresponding row coordinate and the column coordinate of this value are (*i* + 1) and (*j* + 1), respectively. Based on these values, ***a*** and ***b*** can be represented as two-dimensional coordinates ($i_{a}$, $j_{a}$) and ($i_{b}$, $j_{b}$), where $i_{a}$, $j_{a}$ is the row and column coordinate of ***a***, and $i_{b}$, $j_{b}$ is the row and column coordinate of ***b***. ($i_{a}$, $j_{a}$) and ($i_{b}$, $j_{b}$) can be computed from the following formulae:
  (1) $$i_{a}=\left[\frac{a}{N}\right]+1;j_{a}=a\;\mathrm{mod}\;N+1.$$
  (2) $$i_{b}=\left[\frac{b}{N}\right]+1;j_{b}=b\;\mathrm{mod}\;N+1.$$
2. Based on ($i_{a}$, $j_{a}$), we can construct three N-dimensional column vectors for ***a*** as
  (3) $$\tilde{a}=\left[\begin{array}{c} 0^{i_{a}}\\ 1^{N-i_{a}} \end{array}\right],\bar{a}=e_{i_{a}},\hat{a}=\left[\begin{array}{c} 0^{j_{a}-1}\\ 1^{N-j_{a}+1} \end{array}\right],$$
  where $0^{i_{a}}$ denotes an $i_{a}$-dimensional zero vector, $1^{N-i_{a}}$ denotes an $1^{N-i_{a}}$-dimensional vector, and all elements are 1; $e_{i_{a}}$ denotes an N-dimensional unit vector, and the $i_{a}$-th element is 1. In this way, we can obtain the following transformation relation:
  (4) $$\left\{\begin{array}{c} a\leq b\Leftrightarrow \tilde{a}\left[i_{b}\right]+\bar{a}\left[i_{b}\right]*\hat{a}\left[i_{b}\right]=1\\ a>b\Leftrightarrow \tilde{a}\left[i_{b}\right]+\bar{a}\left[i_{b}\right]*\hat{a}\left[i_{b}\right]=0 \end{array}\right.$$
3. Construct $X=[{\tilde{a}}^{T}\;{\bar{a}}^{T}]$, $X^{\prime }=\left[1\;{\hat{a}}^{T}\right]$ and a 2N×(N+1) matrix *Q* satisfying
  (5) $$Q\left[i_{b},1\right]=Q\left[N+i_{b},j_{b}+1\right]=1,$$
  and the other elements in *Q* are 0. We have
  (6) $$\tilde{a}\left[i_{b}\right]+\bar{a}\left[i_{b}\right]*\hat{a}\left[i_{b}\right]=\left[{\tilde{a}}^{T}\;{\bar{a}}^{T}\right]Q\left[\begin{array}{c} 1\\ \hat{a} \end{array}\right]=XQ{X^{\prime }}^{T}.$$

So we have the conclusion that

(7) $$\left\{\begin{array}{c} a\leq b\Leftrightarrow \tilde{a}\left[i_{b}\right]+\bar{a}\left[i_{b}\right]*\hat{a}\left[i_{b}\right]=1\Leftrightarrow XQ{X^{\prime }}^{T}=1\\ a>b\Leftrightarrow \tilde{a}\left[i_{b}\right]+\bar{a}\left[i_{b}\right]*\hat{a}\left[i_{b}\right]=0\Leftrightarrow XQ{X\prime }^{T}=0. \end{array}\right.$$

As we describe above, the judgment on whether data ***a*** is abnormal can be transformed to the equality test of $XQX^{\prime^{T}}$ = 1 or 0. To be specific, if $XQ{X^{\prime }}^{T}$ = 1, it is equivalent to the fact that *a* is less than or equal to *b*, where *b* is the upper limit of the normal value we set. Therefore it means that the data *a* is normal. The opposite is also true.

To help readers better understand the principle, we add a numerical example here. Supposed that N=10,a=55,b=60. We have $i_{a}=\left[\frac{55}{10}\right]+1=6;j_{a}=55\;\mathrm{mod}\;10+1=6$, and $i_{b}=\left[\frac{60}{10}\right]+1=7;j_{b}=60\;\mathrm{mod}\;10+1=1$. Therefore, $\tilde{a}={\left[0000001111\right]}^{T}$, $\bar{a}={\left[0000010000\right]}^{T}$, and $\hat{a}=\left[0000011111\right]$. Further, we can obtain $\tilde{a}\left[7\right]+\bar{a}\left[7\right]*\hat{a}\left[7\right]=1$. Next we construct the matrix $X,X^{\prime }and\;Q$ as described above. Finally, we can obtain that $XQ{X^{\prime }}^{T}=1$, which is also equivalent to a≤b.

## 4. System Model

In this section, we will introduce the system model and the adversary model of the proposed scheme.

### 4.1. System Model

**Figure 2:**
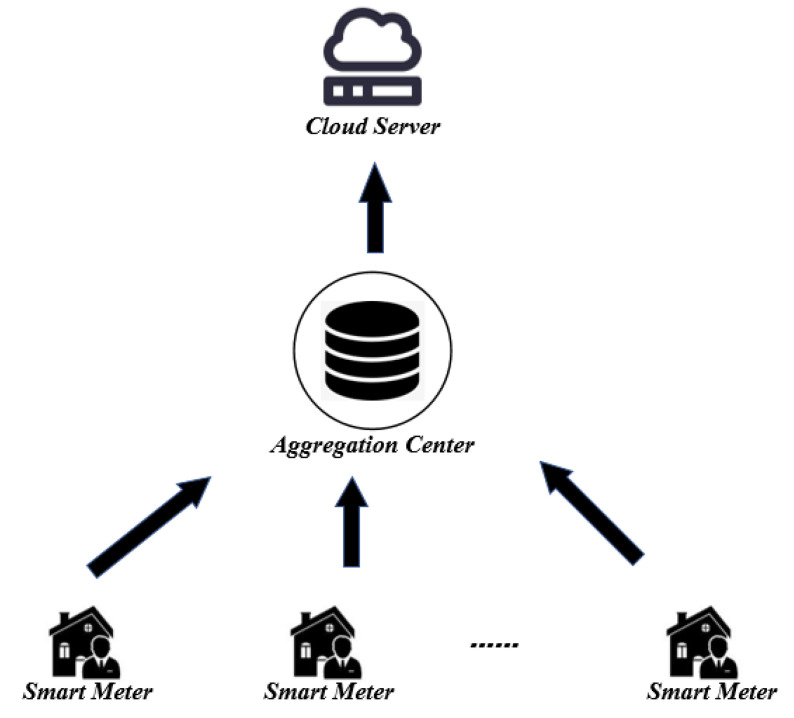
System model.

The system model of our scheme is shown in Figure 2, which consists of three entities: smart meters(*SM*), the aggregation center (*AC*), and the cloud server(*CS*).

- Smart Meters (*SM*): Smart meters are intelligent devices installed at users’ premises with limited computing resources. Each smart meter encrypts the electricity usage data and reports it to the aggregation center.
- Aggregation Center (*AC*): The aggregation center has sufficient computing power to collect and aggregate the electricity usage data reported by the smart meters.
- Cloud Server (*CS*): The cloud server receives and analyzes the aggregated results sent by the *AC*, thus making appropriate production decisions and reasonable electricity distribution.

### 4.2. Adversary Model

In this scheme, we assume that:

- Users may not only try to steal electricity by compromising smart meters, but also be interested in the privacy of other users’ electricity usage data. In addition, there may be cases where the meter fails and reports abnormal electricity consumption data.
- *AC* and *CS* are semi-honest. This means that the two entities will honestly execute the proposed protocol and do not tamper with the computational results, but they may attempt to learn individual electricity usage data as much as possible. Besides, *AC* and *CS* will not collude with each other.
- Any probabilistic polynomial-time adversary can intercept the channels between *SM*s and *AC* and the channels between *AC* and *CS* to obtain the reported data.

Other security issues are beyond the scope of our scheme.

### 4.3. Security Goals and Functionality

On the basis of the system model and adversary model above, our system should satisfy the following security goals and functionality requirements.

- **Data privacy:** Because the data reported by the electricity meters is closely linked to the users’ daily habits and household situations, the proposed scheme should ensure that the privacy of users’ electricity usage data is not compromised by curious internal entities, as well as by external attackers.
- **Filter abnormal data:** In order to prevent the abnormal electricity usage data reported by the electricity meters from affecting the accuracy of the aggregation results, the abnormal electricity usage data should be filtered out during the aggregation process.
- **Trace abnormal source:** The proposed scheme should track the source of abnormal data to further repair and maintain abnormal meters.

## 5. The Proposed Scheme

**Figure 3:**
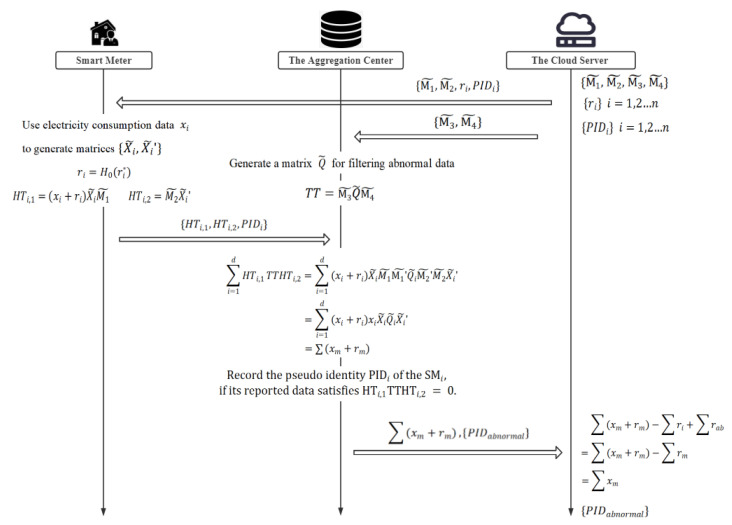
The work flow of our scheme.

Our scheme is mainly composed of five stages: system initialization, registration, data encryption, aggregation and filtering, and decryption. In addition, the work flow of our scheme is presented in Figure 3.

### 5.1. System Initialization

At this stage, the cloud server generates two random non-singular matrices, $\tilde{M_{1}}\in R^{\left(2N+4)\times (2N+4\right)}$ and $\tilde{M_{2}}\in R^{\left(N+5)\times (N+5\right)}$, and computes their inverse matrices, $\tilde{M_{3}}$, $\tilde{M_{4}}$. After that, the public parameters of the system can be denoted as $\tilde{M_{1}}$, $\tilde{M_{2}}$, $\tilde{M_{3}}$, $\tilde{M_{4}}$, and $H_{0}$. Here, the symbol $H_{0}$ is a collision-resistant one-way hash function.

### 5.2. Registration

When the smart meter $SM_{i}$ registers with the cloud server, the cloud server generates a random number $r_{i}$ and a pseudo-identity $PID_{i}$ for it. Then, the cloud server sends, {*PID_I_*,*r_i_*} to it over a secure channel.

### 5.3. User: Data Encryption

(1) For electricity usage data $x_{i}$, the smart meter $SM_{i}$ generates two random numbers, $\mu_{x,i}$ and $\mu_{x,i}^{\prime }$, and constructs the following matrices $\left\{\tilde{X_{i}},\tilde{X}{}_{i}^{\prime }\right\}$ as

(8) $$\tilde{x_{i}}=\left[\begin{array}{c} 0^{i_{x}}\\ 1^{N-i_{x}} \end{array}\right],\bar{x_{i}}=e_{i_{x}},\hat{x_{i}}=\left[\begin{array}{c} 0^{j_{x}-1}\\ 1^{N-j_{x}+1} \end{array}\right],$$

where $\tilde{x_{i}}$, $\bar{x_{i}}$, and $\hat{x_{i}}$ are constructed as in Section 3, i.e.:

(9) $$X_{i}=\left[{{\tilde{x}}_{i}}^{T}\;{{\bar{x}}_{i}}^{T}\right],X{{}^{\prime }}_{i}=\left[1\;{{\hat{x}}_{i}}^{T}\right]$$

(10) $${\tilde{X}}_{i}=\left[\left(x_{i}+r_{i}\right)X_{i}\;R_{x,i}\;1\;0\right]$$

(11) $$\tilde{X}{{}^{\prime }}_{i}=\left[\begin{array}{c} {X_{i}^{\prime }}^{T}\\ R{}_{x,i}^{\prime }\\ 0\\ 1 \end{array}\right],$$

where $R_{x,i}=\left[\mu_{x,i}\;\mu_{x,i}\right],R_{x,i}^{\prime }=\left[\mu^{\prime }{}_{x,i}\;\mu^{\prime }{}_{x,i}\right]$.

(2) The smart meter $SM_{i}$ encrypts $\left\{\tilde{X_{i}},\tilde{X}{}_{i}^{\prime }\right\}$ into the ciphertext $\left\{{HT}_{i,1},{HT}_{i,2}\right\}$ as follows:

(12) $$HT_{i,1}={\tilde{X}}_{i}{\tilde{M}}_{1},\;HT_{i,2}={\tilde{M}}_{2}\tilde{X}{}_{i}^{\prime }.$$

(3) Finally, the smart meter $SM_{i}$ reports the ciphertext $\left\{HT_{i,1},HT_{i,2},PID_{i}\right\}$ to the aggregation center.

### 5.4. The Aggregation Center: Aggregation and Filtering

(1) The aggregation center generates the matrix $\tilde{Q}$ according to the upper limit of normal data, ***q***:

(13) $$\tilde{Q}=\left[\begin{array}{cccc} Q & 0 & 0 & 0\\ 0 & R_{Q} & 0 & 0\\ 0 & 0 & \mu_{Q,1} & 0\\ 0 & 0 & 0 & \mu_{Q,2} \end{array}\right],$$

where $\mu_{Q,1}$ and $\mu_{Q,2}$ are random numbers, and Q is a 2N×(N+1) matrix constructed as in Section 3:

(14) $$R_{Q}=\left[\begin{array}{cc} r_{Q,1} & r_{Q,2}\\ r_{Q,3} & -(r_{Q,1}+r_{Q,2}+r_{Q,3}) \end{array}\right],$$

where $r_{Q,1}$, $r_{Q,1}$, and $r_{Q,1}$ are random numbers.

Then, the aggregation center constructs matrix *TT* according to the matrix $\tilde{Q}$ and the matrix $\left\{{\tilde{M}}_{3},{\tilde{M}}_{4}\right\}$ as in the following equation:

(15) $$TT={\tilde{M}}_{3}\tilde{Q}{\tilde{M}}_{4}.$$

(2) The aggregation center aggregates the reported data to obtain the aggregation result $R^{\prime }$ according to the following equation:

(16) $$\begin{array}{cc} R^{\prime } & =\sum_{i=1}^{n}HT_{i,1}TTHT_{i,2}\\ & =\sum_{i=1}^{n}\left(x_{i}+r_{i}\right){\tilde{X}}_{i}{\tilde{M}}_{1}{\tilde{M}}_{3}{\tilde{Q}}_{i}{\tilde{M}}_{4}{\tilde{M}}_{2}{{\tilde{X}}_{i}}^{\prime }\\ & =\sum_{i=1}^{n}\left(x_{i}+r_{i}\right){\tilde{X}}_{i}{\tilde{Q}}_{i}{{\tilde{X}}_{i}}^{\prime }\\ & =\sum_{i=1}^{n}\left(x_{i}+r_{i}\right)\left(XQ{X^{\prime }}^{T}+{R^{\prime }}_{x,i}R_{Q}R_{x,i}\right)\\ & =\sum_{i=1}^{n}\left(x_{i}+r_{i}\right)XQ{X^{\prime }}^{T}. \end{array}$$

For abnormal data, the result of $XQ{X^{\prime }}^{T}$ is 0, therefore the result of the formula $HT_{i,1}TTHT_{i,2}$ is 0. While, for normal data, $XQ{X^{\prime }}^{T}=1$, the result of the formula $HT_{i,1}TTHT_{i,2}$ is still $\left(x_{i}+r_{i}\right)$. In this way, the abnormal data is automatically filtered in the process of aggregation, that is, the aggregation result $R^{\prime }$ is $\sum \left(x_{m}+r_{m}\right)$, where $x_{m}$ represents the normal electricity usage data, and $r_{m}$ represents its corresponding masking value. Besides, if reported data are judged to be abnormal, the aggregation center will record their source, $PID_{ab}$, and send it to the cloud server.

(17) $$\left(x_{i}+r_{i}\right)XQ{X^{\prime }}^{T}=\left\{\begin{array}{c} \left(x_{i}+r_{i}\right),if\;data\;\;x_{i}\;\;is\;normal\\ 0,otherwise \end{array}\right.$$

(3) Lastly, the aggregation center sends the aggregated result, $R^{\prime }=\sum \left(x_{m}+r_{m}\right)$, and the pseudo identities, $\left\{PID{}_{ab}\right\}$, of the abnormal smart meters to the cloud server.

### 5.5. The Cloud Server: Decryption

After receiving the aggregated result, $R^{\prime }=\sum \left(x_{m}+r_{m}\right)$, and the pseudo identities, $\left\{PID_{ab}\right\}$, of the abnormal smart meters from the aggregation center, the cloud server decrypts the data to obtain the real aggregated result R as in the following equation:

(18) $$\begin{array}{cc} R\; & =\sum \left(x_{m}+r_{m}\right)-\left(\sum r_{m}\right)\\ \; & =\sum \left(x_{m}+r_{m}\right)-\left(\sum r_{i}-\sum r_{ab}\right)\\ \; & =\sum x_{m}, \end{array}$$

where $r_{ab}$ represents the masking value corresponding to the smart meter which reports abnormal data.

Therefore, the cloud server can obtain the accurate aggregated result *R* that does not include abnormal data and the pseudo identities $PID_{ab}$ of abnormal meters, so that it can make appropriate production decisions and check for abnormal smart meters.

As the range of electricity usage data expands, the constructed matrix will become larger, which greatly increases the communication cost. For example, the bit length of the report data will be at least 1000 bits when the electricity usage data reaches 1000.

To solve this problem, a mapping function $f:S\to S^{*}$ is proposed to map the original data to a smaller set, where *S* and $S^{*}$ are the original data set and the mapped set, respectively. For any $x_{i}\in S$, there exists a unique ${x_{i}}^{*}\in S^{*}$ corresponding to it and ${x_{i}}^{*}=\left[x_{i}/b\right]$, where *b* is determined by the filtering accuracy. By sacrificing some accuracy within an acceptable range, communication overheads can be greatly reduced.

## 6. Security Analysis

In this section, we present the security proof of the proposed scheme to solve the problem of adversarial models.

**Theorem** **2.** *(Resistant to the middle-man attack) The proposed scheme can ensure that the privacy of users’ data is not compromised by the external adversaries.*

**Proof.**  The confidentiality of users’ electricity data $x_{i}$(i=1,2,…,n) and the aggregation result $\sum x_{m}$ will be proved below.

If the PPT adversary tries to obtain $x_{i}$ from $\left\{HT_{i,1},\;HT_{i,2}\right\}$, (s)he must know $r_{i}$ since $HT_{i,1}=\left(x_{i}+r_{i}\right){\tilde{X}}_{i}{\tilde{M}}_{1}$ and $HT_{i,2}={\tilde{M}}_{2}{{\tilde{X}}_{i}}^{\prime }$. However, $r_{i}$ is a random number only available to registered users and the cloud server. Consequently, the external adversaries cannot infer the individual electricity data $x_{i}$ from $\left\{HT_{i,1},\;HT_{i,2}\right\}$.

If the external adversary tries to derive $\sum x_{m}$ from *R*, (s)he needs to know the sum of random numbers $\sum r_{m}$ since *R* =$\sum \left(x_{m}+r_{m}\right)$. However, $\sum r_{m}$ is only available to the cloud server. Thus, adversaries cannot infer the normal total electricity usage data $\sum x_{m}$.

To sum up, any adversary cannot recover individual electricity usage data $x_{i}$ or total electricity usage data $\sum x_{m}$ that excludes abnormal data. □

**Theorem** **2.** *Our proposed scheme can achieve the privacy of data transmitted by a smart meter.*

**Proof.**  In our scheme, the attackers of data privacy can be divided into two categories: internal attackers and external attackers. For external attacks, they can be resisted, since an encryption algorithm is adopted in our scheme. For internal attackers, we discuss it in the following three cases. □

1. When the internal attacker is the aggregation center, although it can obtain the encrypted users’ electricity usage data, it cannot gain the users’ real electricity usage data. Specifically, the aggregation center can get $\left\{HT_{i,1},HT_{i,2}\right\}$ reported by smart meters, where $HT_{i,1}=\left(x_{i}+r_{i}\right){\tilde{X}}_{i}{\tilde{M}}_{1}$, and $HT_{i,2}={\tilde{M}}_{2}{{\tilde{X}}_{i}}^{\prime }$. If the aggregation center tries to recover $x_{i}$ from $\left\{HT_{i,1},HT_{i,2}\right\}$, it must know $r_{i}$. However, $r_{i}$ is only available to the user *i* and the cloud server. Therefore, the proposed scheme can resist privacy attacks on the transmitted data from the aggregation center.
2. When the internal attacker is the cloud server, although it can obtain the aggregated result of normal electricity usage data, it cannot gain the electricity usage data of a single user. Concretely, the cloud server can only obtain $\sum (x_{m}+r_{m})$ from the aggregation center, that is, it can only obtain the aggregated result of normal electricity usage data $\sum x_{m}$, which is computed by $\sum (x_{m}+r_{m})-\sum r_{m}$. Therefore, the proposed scheme can resist privacy attacks on the transmitted data from the cloud server.
3. When the internal attacker is a valid smart meter. Although it can intercept electricity usage data reported by other smart meters, it cannot obtain that the corresponding user’s true electricity usage information $x_{i}$, because the masking value $r_{i}$ is known only to the corresponding user and the cloud server. Hence, any smart meter cannot recover the electricity usage data of other smart meters.

To sum up, our scheme can achieve data privacy.

**Theorem** **3.** *It is infeasible to learn users’ electricity usage data information according to the reported data in different rounds.*

**Proof.**  In each round of data aggregation, the smart meter $SM_{i}$ updates the masking value $r_{i}$ as $r_{i}^{\prime }=H_{0}\left(r_{i}\right)$. Even if the adversary gets the reported data in two different rounds, $\left(x_{i}+r_{i}\right)$ and $\left(x{}_{i}^{\prime }+r{}_{i}^{\prime }\right)$, (s)he can only obtain $\left(x{}_{i}^{\prime }+r{}_{i}^{\prime }\right)-\left(x_{i}+r_{i}\right)$, which does not reveal the changes in electricity usage data in the two aggregation rounds. Therefore, it is still infeasible to obtain information related to users’ electricity usage data according to the reported data in different rounds. □

Finally, we compare the security features of our proposed approach with homomorphic encryption schemes [10] and masking-value-based schemes [22,26,27,28]. As shown in Table 2, our scheme has the most comprehensive security functions and features.

## 7. Performance Analysis

In this section, we evaluate the performance of our scheme and compare our scheme with two representative and related schemes, the LCEDA scheme by Su et al. [20] and the DMDA scheme by Song et al. [25]. All of these schemes involve the use of masking values to encrypt the electricity usage data, and our scheme uses matrix encryption to filter abnormal data beyond that. Hence, we primarily evaluate the performance of the proposed scheme with LCEDA and DMDA in terms of communication and computation costs. Table 3 lists some notations for the performance comparisons.

### 7.1. Communication Costs

The communication costs of the LCEDA, the DMDA, and our scheme in the enrollment stage are shown in Table 4. The highest communication costs are mainly concentrated between the cloud server and the smart meters in these schemes.

It costs $\left|Z_{p}\right|+\left|ID\right|$ communication overheads for the aggregation center to register at the cloud server in LCEDA. In addition, each smart meter spends $t\left|Z_{p}\right|+\left|ID\right|$ and $\left|Z_{p}\right|$ on registering at the cloud server and the aggregation center, respectively. Hence, in the enrollment stage, the complexity of communication times in LCEDA is O(1), and the total costs are $\left(t+2\right)\left|Z_{p}\right|+2\left|ID\right|$. In DMDA, the complexity of communication times is O(1), and the aggregation center spends $\left|G\right|$ communication overheads on registering at the cloud server to obtain the mask values, while the smarts register spends $\left(t+2\right)\left|Z_{p}\right|+\left|G\right|+2\left|ID\right|$ communication overheads. Therefore, the total length of a communication message is constant in the enrollment stage of DMDA. In our scheme, the complexity of communication times is O(1), and it costs $\left|M_{1}\right|+\left|M_{2}\right|+\left|Z_{p}\right|+\left|ID\right|$ communication overheads for the smart meters to register at the cloud server.

To sum up, in the enrollment stage, the total length of the communication message of LCEDA in the enrollment stage is linear with *t*, hence, it has the highest communication costs among these schemes. Although both the DMDA and our scheme are constant, our scheme is less efficient than DMDA, comprehensively considering communication times and message length.

### 7.2. Computation Costs

To evaluate performance, we conducted some experiments on a computer running Windows 10 with a 3.00 GHz Intel Core i5-8500 CPU and 8 GB memory. These experiments were run separately 50 times to obtain the mean results using the GNU Multiple Precision Arithmetic (GMP) Library and Pairing-Based Cryptography (PBC) Library.

**Figure 4:**
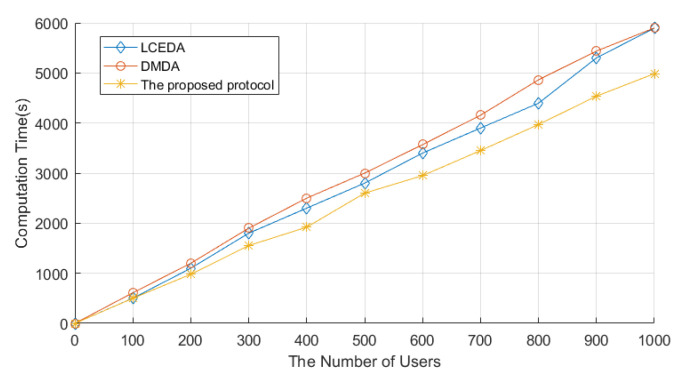
Computation Time of Enrollment Stage. The figure shows how the time required for LCEDA, DMDA, and the proposed scheme in the enrollment stage changes as the number of users increases. In addition, the figure reflects that the proposed scheme requires relatively little time compared to the other two.

The system initialization stage consists of two stages: the system setup stage and the enrollment stage. We set the number of users as 1000 in the implementation. The system setup stage in LCEDA, DMDA, and our scheme costed 8.74 ms, 29.9 ms, and 4.90 ms, respectively. The comparison of computation costs related to LCEDA, DMDA, and our scheme in the enrollment stage is shown in Figure 4, where we set the number of users to vary from 100 to 1000 at an increasing interval of 100. In LCEDA, the smart meters spent $\left(t+1\right)T_{(t-1)-poly}$ on registering at the cloud server and the aggregation center without negotiating with each other. As shown in Figure 4, the computation time of LCEDA ranged from 502.2 ms to 5895.2 ms when the number of users varied from 100 to 1000. The computation costs of DMDA are $2(T_{pm}+T_{h}+T_{a})$. In our scheme, the smart meters and the aggregation center register at the cloud server, which costs $4M_{m}$, and the computation time of the proposed protocol ranges from 465.3 ms to 4897.6 ms.

**Figure 5:**
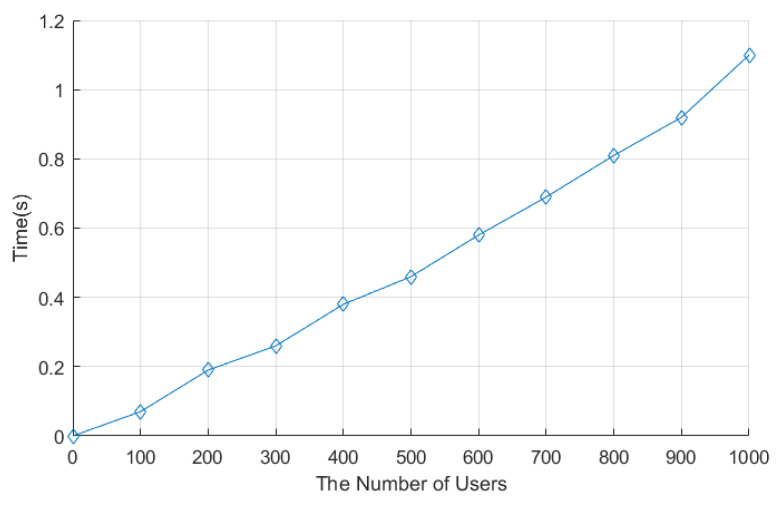
Time to encrypt data in the proposed protocol.

**Figure 6:**
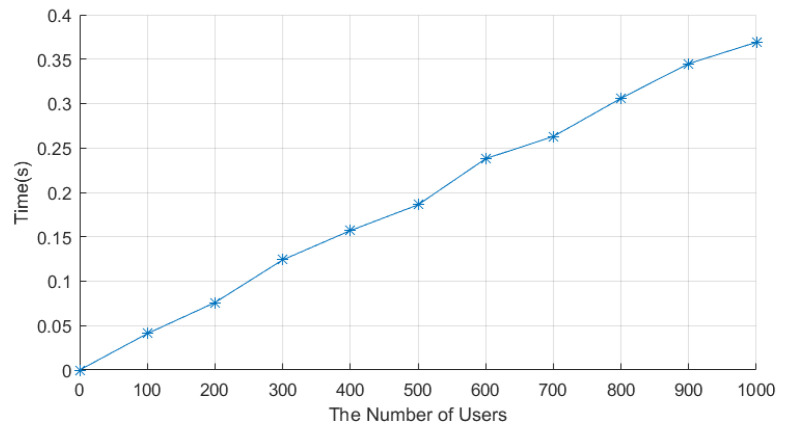
Time to aggregate data in the proposed protocol.

The data collection stage consists of three stages: the data encryption stage, the aggregation stage, and the decryption stage. The encryption times of LCEDA, DMDA, and our scheme are shown in Table 5. Each smart meter in LCEDA needed 0.001 ms to encrypt the electricity usage data, while our scheme needed 0.3 ms to encrypt. The aggregation of the encrypted electricity usage data costs 28.3 ms, 28.3, and 33.2 ms in LCEDA, DMDA, and our scheme, respectively, when the number of users is 1000. In LCEDA and DMDA, the cloud center needs 0.53 ms to decrypt the aggregation result, whereas our scheme only needs 0.13 ms. Finally, the time to encrypt data and to aggregate data in our scheme are shown in Figure 5 and Figure 6, respectively. Therefore, LECDA and DMDA have lower computation costs $\left(2\left(T_{pm}+T_{h}\right)+\left(n+1\right)T_{a}+T_{s}\right)$ and $\left({\left(n+t\right)}_{T(t-1)-ploy}+2\left(n-1\right)T_{m}+\left(2n+1\right)T_{a}+T_{s}\right)$, respectively, compared to our scheme, which is because they do not involve filtering abnormal electricity usage data and do not support finding out the source of abnormal electricity usage data.

To sum up, our scheme needs to pay more computation costs for filtering abnormal users and finding out the source of abnormal users, but the increase is not significant, that is, our scheme is indeed efficient.

## 8. Conclusions

In this paper, we propose a lightweight and privacy-friendly data aggregation scheme against abnormal data to solve the problem that the abnormal electricity usage data cannot be filtered out when it is encrypted. Besides, our scheme can find out the smart meters which reported the abnormal data. Compared with other complex schemes, our scheme only uses a lightweight matrix encryption, which has lower computational costs and is more suitable for smart meters with limited computing capacity. Finally, a security analysis of our proposed scheme is presented to prove that our scheme can fully protect the privacy of users’ electricity usage data. In addition, the performance evaluations and experiments validate the effectiveness and practicability of our scheme. Consequently, our scheme can be implemented in smart grids to effectively filter abnormal data and find out its source.

It is hard to say that our scheme has no drawbacks. We mainly focus on filtering abnormal data during aggregation and finding the source of the abnormal data. We use lightweight matrix encryption to process real-time electricity usage data. However, as the range of electricity usage data expands, the constructed matrix will become larger, which will gradually increase the computational and communication overheads. To overcome this problem, we mapped the original data onto a smaller data set to reduce the size of the construction matrix, and the mapping function was determined by the filtering accuracy. By sacrificing some accuracy within an acceptable range, communication overheads can be greatly reduced. In future work, we will focus on reducing computing and communication overheads while ensuring better filtering accuracy. 

## Figures and Tables

**Table 1:** Security feature comparisons.

Scheme	Data Confidentiality	Resistance to Middle-Man Attacks	Filtering of Abnormal Data	Tracing the Source of Abnormal Data	Computational Cost
[10,11,12]	✓	✓	×	×	High
[13,14]	✓	✓	×	×	High
[15,16]	✓	✓	×	×	Low
[17]	✓	✓	×	×	High
[18,19,20]	✓	✓	×	×	High
[21,22]	✓	✓	×	×	Low
[23]	✓	✓	×	×	Low
[24]	✓	✓	✓	✓	High
The proposed protocol	✓	✓	✓	✓	Low

**Table 2:** Security feature comparisons.

Scheme	Data Confidentiality	Resistance against Man-in-the-Middle Attacks	Forward/Backward Secrecies	Filtering of Abnormal Data	Finding the Source of Abnormal Data
AMDA [10]	✓	×	×	×	×
DMDA [26]	✓	✓	×	×	×
LPSDA [21]	✓	×	×	×	×
ESPDA [27]	✓	✓	×	×	×
ERDA [28]	✓	×	✓	×	×
LCEDA [22]	✓	✓	✓	×	×
The proposed protocol	✓	✓	✓	✓	✓

**Table 3:** Notations.

Notation	Semantics	Notation	Semantics
$T_{(t-1)-poly}$	Time of evaluation operation of a (t−1)-polynomial	$\left|Z_{p}\right|$	Element size in $Z_{p}$
$\left|ID\right|$	Bit length of the identifier	$\left|G\right|$	Element size in *G*
$\left|M_{1}\right|$	(2N+4)×(2N+4) Matrix size	$\left|M_{3}\right|$	1×(2N+4) Matrix size
$\left|M_{2}\right|$	(N+5)×(N+5) Matrix size	$\left|M_{4}\right|$	(N+5)×1 Matrix size
$T_{a}$	Time of an addition operation in $Z_{p}$	$\left|T_{h}\right|$	Time of a hash operation
$T_{s}$	Time of a subtraction operation in $Z_{p}$	$\left|T_{pm}\right|$	Time of a point multiplication operation
$M_{m}$	Time of a multiplication operation in matrix	$\left|M_{a}\right|$	Time of an addition operation in matrix

**Table 4:** Comparisons of communication costs in the enrollment stage.

Scheme	CS↔SM	AC↔SM	CS↔AC	Total Costs
LCEDA	$t\left|Z_{p}\right|+\left|ID\right|$	$\left|Z_{p}\right|$	$\left|Z_{p}\right|+\left|ID\right|$	$\left(t+2\right)\left|Z_{p}\right|+2\left|ID\right|$
DMDA	$2\left|Z_{p}\right|+\left|G\right|+2\left|ID\right|$	−	$\left|G\right|$	$2(\left|Z_{p}\right|+\left|G\right|+\left|ID\right|)$
The proposed protocol	$\left|M_{1}\right|+\left|M_{2}\right|+\left|Z_{p}\right|+\left|ID\right|$	−	$\left|M_{3}\right|+\left|M_{4}\right|$	$\left|M_{1}\right|+\left|M_{2}\right|+\left|Z_{p}\right|+\left|ID\right|+\left|M_{3}\right|+\left|M_{4}\right|$

**Table 5:** Comparisons of computation costs.

Scheme (*n* = 1000)	Encryption		Aggregation		Decryption	
	Times (ms)	Costs	Times (ms)	Costs	Times (ms)	Costs
LCEDA	$T_{a}$	0.001	$\left(n-1\right)T_{a}$	28.3	$T_{s}$	0.53
DMDA	$T_{a}$	0.001	$\left(n-1\right)T_{a}$	28.3	$T_{s}$	0.53
The proposed protocol	$T_{a}+2M_{m}$	0.1	$2nM_{m}+\left(n-1\right)T_{a}$	33.2	$T_{s}$	0.13

## Data Availability

The datasets generated during and/or analysed during the current study are available from the corresponding author on reasonable request.

## Abbreviations

The following abbreviations are used in this manuscript:

SM	smart meter
AC	aggregation center
CS	cloud server
